# Role of the Fractalkine Receptor in CNS Autoimmune Inflammation: New Approach Utilizing a Mouse Model Expressing the Human CX3CR1^I249/M280^ Variant

**DOI:** 10.3389/fncel.2018.00365

**Published:** 2018-10-17

**Authors:** Sandra M. Cardona, Sangwon V. Kim, Kaira A. Church, Vanessa O. Torres, Ian A. Cleary, Andrew S. Mendiola, Stephen P. Saville, Stephanie S. Watowich, Jan Parker-Thornburg, Alejandro Soto-Ospina, Pedronel Araque, Richard M. Ransohoff, Astrid E. Cardona

**Affiliations:** ^1^Department of Biology, The University of Texas at San Antonio, San Antonio, TX, United States; ^2^South Texas Center for Emerging Infectious Diseases, The University of Texas at San Antonio, San Antonio, TX, United States; ^3^Department of Microbiology and Immunology, Sidney Kimmel Medical College of Thomas Jefferson University, Philadelphia, PA, United States; ^4^Department of Neurology and Neurotherapeutics, University of Texas Southwestern Medical Center, Dallas, TX, United States; ^5^Department of Biomedical Sciences, Grand Valley State University, Allendale, MI, United States; ^6^Gladstone Institute of Neurological Disease, San Francisco, CA, United States; ^7^Department of Immunology, Center for Inflammation and Cancer, The University of Texas MD Anderson Cancer Center, Houston, TX, United States; ^8^Department of Genetics, The University of Texas MD Anderson Cancer Center, Houston, TX, United States; ^9^Basic Sciences Department, Research and Innovation in Chemical Formulations, University EIA, Envigado, Colombia; ^10^Third Rock Ventures, Boston, MA, United States

**Keywords:** EAE (experimental autoimmune encephalitis), fractalkine receptor, microglia, multiple sclerois and neuroimmunology, demyelination and neuronal damage

## Abstract

Multiple sclerosis (MS), an inflammatory demyelinating disease of the central nervous system (CNS) is the leading cause of non-traumatic neurological disability in young adults. Immune mediated destruction of myelin and oligodendrocytes is considered the primary pathology of MS, but progressive axonal loss is the major cause of neurological disability. In an effort to understand microglia function during CNS inflammation, our laboratory focuses on the fractalkine/CX3CR1 signaling as a regulator of microglia neurotoxicity in various models of neurodegeneration. Fractalkine (FKN) is a transmembrane chemokine expressed in the CNS by neurons and signals through its unique receptor CX3CR1 present in microglia. During experimental autoimmune encephalomyelitis (EAE), CX3CR1 deficiency confers exacerbated disease defined by severe inflammation and neuronal loss. The CX3CR1 human polymorphism I249/M280 present in ∼20% of the population exhibits reduced adhesion for FKN conferring defective signaling whose role in microglia function and influence on neurons during MS remains unsolved. The aim of this study is to assess the effect of weaker signaling through hCX3CR1^I249/M280^ during EAE. We hypothesize that dysregulated microglial responses due to impaired CX3CR1 signaling enhance neuronal/axonal damage. We generated an animal model replacing the mouse CX3CR1 locus for the hCX3CR1^I249/M280^ variant. Upon EAE induction, these mice exhibited exacerbated EAE correlating with severe inflammation and neuronal loss. We also observed that mice with aberrant CX3CR1 signaling are unable to produce FKN and ciliary neurotrophic factor during EAE in contrast to wild type mice. Our results provide validation of defective function of the hCX3CR1^I249/M280^ variant and the foundation to broaden the understanding of microglia dysfunction during neuroinflammation.

## Introduction

Inflammatory demyelination is a hallmark of the pathology of multiple sclerosis (MS) and microglia are considered responsible for exacerbating myelin loss. Although the link between microglia mediated inflammation and neurodegeneration is being increasingly recognized, the mechanisms that connect microglia, inflammation, and demyelination are unclear. Our previous studies have shown that the neuronal derived chemokine, fractalkine (FKN, also formally known as CX3CL1), and its unique receptor, CX3CR1, expressed by microglia, are highly abundant in the healthy brain and spinal cord and have direct roles in inhibiting microglia inflammatory behavior (Cardona et al., 2006). Notably, demyelination and neuronal damage are exacerbated in mice lacking CX3CR1 (Garcia et al., 2013) suggesting that microglia exert anti-inflammatory roles during demyelination. In humans, two single nucleotide polymorphisms in the CX3CR1 locus produce receptors with proposed defective binding to FKN and appear to play a deleterious role in MS progression. Most individuals carry the CX3CR1^V 249/T280^ allele, while the variant CX3CR1^I249/M280^ is present in 25–30% of the population. Cells expressing CX3CR1^I249/M280^ are less responsive to FKN (McDermott et al., 2003) and in a small cohort of patients, the CX3CR1^M280^ mutation was found preferentially associated with secondary progressive MS in which patients develop more demyelinated lesions (Stojkovic et al., 2012). However, the role of the human variant CX3CR1^M280^ in microglia distribution, in modulating central nervous system (CNS) inflammation, and demyelination in MS models has not been directly addressed.

Due to the proposed defect of the human CX3CR1^I249/M280^ variant in signaling to FKN, we sought to determine how closely the expression of the human variant recapitulates the phenotype observed in CX3CR1-deficient mice. For this, we generated a model in which mice in the C57BL6 background express the I249/M280 variant of the human CX3CR1 receptor *in lieu* of the murine counterpart. In this study, we report the phenotype of CX3CR1^I249/M280^ expressing mice under active disease in myelin oligodendrocyte glycoprotein (MOG_35-55_) induced experimental autoimmune encephalomyelitis (EAE). We describe disease progression, CNS cellular infiltration, demyelination, neuronal loss, and expression of inflammation-associated genes. Our results show that EAE progression is more severe in CX3CR1^I249/M280^ mice than WT mice and closely resembles the disease course observed in CX3CR1-KO mice. More severe demyelination and neuronal loss was also observed in CX3CR1^I249/M280^ mice, particularly in cerebellar regions. Differences in disease severity did not appear to be associated with increased frequencies of IL-17 or IFN-γ producing MOG_35-55_ specific T cells, as revealed by comparable proportions of IFN-γ and IL-17 producing T cells in WT, CX3CR1-KO and CX3CR1^I249/M280^ draining lymph nodes at 11 days post-immunization. CD4^+^ T cells represented the predominant population expressing IL-17 or IFN-γ, with negligible detection of IL-17/IFN-γ double positive cells in intracellular cytokine image flow cytometry assays. In CNS tissues, analyses of inflammatory cells revealed comparable levels of infiltration of dendritic cells (DCs), neutrophils and CD4^+^ T cells, however, CX3CR1^I249/M280^ mice displayed increased frequencies of CD45^Lo^/CD11b^+^ microglia and CD8^+^ T cells. Transcriptional analyses revealed several genes highly upregulated in all genotypes but of particular interest was lipocalin 2, and immunostaining confirmed its significant upregulation in WT tissues compared to CX3CR1-KO and CX3CR1^I249/M280^ brains. Overall these results indicate that CX3CR1^I249/M280^ expressing mice exhibit an EAE phenotype that closely resembles the phenotype of CX3CR1-KO mice. These findings highlight the neuroprotective effects of FKN during EAE and provide a potential novel pathway to understand how LCN2 could be targeted to control inflammation and promote repair, based on its regulation by CX3CR1/FKN signaling.

## Materials and Methods

### Chemotactic Assay

Responsiveness of human CX3CR1 receptors to mouse fractalkine was tested *in vitro* using bone marrow derived macrophages grown on poly-D-lysin coated flasks in complete RPMI media (20% FBS, 2 mM glutamine, 100 μg/mL streptomycin, 100 U/mL penicillin, and 40 ng/mL rhu-M-CSF) for 4 days and electroporated with 2 μg of pIRES2 expression plasmid containing the human CX3CR1 ORF for the reference hCX3CR1^V 249/TM280^ or hCX3CR1^I249/M280^ genes. Cells were expanded for 3 days in complete media and the number of cells that mobilized across a millicell PCF 8 μm insert were quantified by virtue of RFP expression from expressing vector and DAPI nuclear staining. Five images at 20x were acquired per insert and cells were quantified. Data was reported as number of cells per mm^2^.

### Molecular Cloning and Generation of hCX3CR1^I249/M280^ Mice

Genomic fragments of the murine CX3CR1 locus were isolated from the pEasyFlox-CX3CR1-eGFP targeting vector (Jung et al., 2000) by PCR to construct a targeting vector on the pEasyFLox backbone containing the 5′ untranslated region [short arm (SA)] of mouse CX3CR1, variant CX3CR1^I249/M280^ complete open reading frame (ORF), a neomycin gene within lox P sites, and the 3′ untranslated region of mouse CX3CR1 [long arm (LA)]. A genomic fragment containing the hCX3CR1^I249/M280^ ORF was subcloned from plasmid pSC59 (McDermott et al., 2003). The homologous regions of the final vector consisted of a PCR amplified 1.2 Kb fragment (SA) immediately upstream of the hCX3CR1 start codon, a 7.0 Kb fragment spanning the 3′ mouse CX3CR1 UTR (LA), and selection markers of the pEasyFlox cloning vector. Hybrid C57BL/6–129 embryonic stem cells were expanded and electroporated with the NotI linearized vectors (The University of Texas MD Anderson Cancer Center Genetically Engineered Mouse Facility). After selection with G418 and FIAU, surviving clones were grown and DNA was isolated. Southern blots were used to confirm homologous recombination at 5′ and 3′ end utilizing probes specific for mouse genome loci (SAprobe3 and LAprobe 3). Germ line transmission yielded hCX3CR1^I249/M280^ mice that were backcrossed (>N6) to C57BL/6 mice. Cx3cr1 transcript levels were assessed in microglia sorted from WT, KO, and hCX3CR1^I249/M280^ mice. Mice were genotyped by polymerase chain reaction (PCR) using specific primers to identify not only insertion of the human cx3cr1 gene but also locus specific insertion: SA-13: 5′-CCAAGACAGAATTTCACTGTGTAGC-3′; SA-14: 5′-CAACAAATTTCCCACCAGGCCAATG-3′; SA-15: 5′-TACCTGGCCATCGTCCTGGCCGCCA-3′; SA-16: 5′-ACGAGACTAGTGAGACGTGCTACTT-3′. Crossing of these mice with B6.FVB-TG (EIIa-cre)C5379Lmgd/J (The Jackson Laboratory, Stock No. 003724) resulted in cre-mediated recombination of the floxed neomycin resistance gene, removal of the stop cassette, and concomitant loss of the SA-15/SA-16 1,004 bp fragment and subsequent expression of the hCX3CR1 unit. Validation of the introduction of the human gene and respective I249/M280 mutation was further confirmed by sequencing.

### Mice

C57BL/6J wild type (WT) and *CX3CR1^GFP/GFP^* mice were purchased from The Jackson laboratory (Bar Harbor, ME, United States), hCX3CR1^I249/M280^ were backcrossed to C57BL/6J background for seven generations. Mice were maintained at the Laboratory Animal Resource Center at the University of Texas at San Antonio. All experiments were performed in accordance with NIH guidelines and approved by UTSA-Institutional Animal Care and Use Committee.

### Induction of Active EAE

Active EAE was induced in female mice 8–10 weeks old by subcutaneous immunization with 100 μg of MOG_35-55_ peptide in CFA, and 200 ng of pertussis toxin on days 0 and 2 post-immunization as previously described (Garcia et al., 2013). Mice were weighed and examined daily for EAE signs and scored according to the following scale: 0: no sign of disease, 1: lack of tail tone, 2: abnormal gait and/or hind limb weakness, 2.5: partial hindlimb paralysis, 3: complete hindlimb paralysis, 3.5: ascending paralysis, 4: tetraplegia, and 5: death. Mice were sacrificed when they reached the highest disease score (peak of disease, 16–21 d.p.i) or 2–3 weeks after peak (chronic phase).

### Antibodies

For immunohistochemistry experiments, tissues were stained with primary antibodies directed to identify microglia (rabbit anti-Iba-1, 1:4000, Wako or mouse anti-Iba-1 kindly provided by Dr. Bruce Trapp), inflammatory monocytes (rat anti-CD45, 1:2000, Bio-Rad), cerebellar neurons (rabbit anti-calbindin, 1:1000, Cell signaling Technology), pro-inflammatory cytokine interleukin-1 beta (rabbit anti-IL1 beta, 1:200, abcam), myelin basic protein (MBP) (rabbit anti-MBP, 1:2000, Invitrogen), and astrocytes (rat anti-GFAP, 1:4000, Invitrogen). Secondary antibodies were purchased from Jackson ImmunoResearch Laboratory as follows: Cy3-goat anti rabbit, Cy5-donkey anti rat, Cy2-goat anti mouse, biotinylated-goat anti rabbit, biotinylated-goat anti mouse, and biotinylated-rabbit anti rat. Biotinylated antibodies are detected by using stable DAB (Thermo Fisher Scientific) containing diaminobenzidine and hydrogen peroxidase.

### Immunohistochemistry Staining of CNS Tissues

Mice were placed under deep anesthesia with 5% isoflurane in an induction chamber and sacrificed by perfusion with ice cold Hanks balanced salt solution (HBSS w/o Ca^++^, Mg^++^, Lonza) followed by 4% PFA. Dissected brains were post-fixed overnight in 4% PFA and cryoprotected in 20% glycerol in 80mM phosphate buffer (pH 7.6) for at least 24 h. Free floating sections (30 μm) were generated using a freezing microtome and stored at -20°C until use. For immunostaining, tissues were blocked and permeabilized with 10% normal goat or donkey serum (Jackson ImmunoResearch) containing 0.3% Triton X-100 (Sigma) for 1 h at room temperature. Primary antibodies were incubated overnight at 4°C. Tissues were washed in PBS supplemented with 0.1% Triton X-100 and incubated with host-appropriate secondary antibodies for 2 h at room temperature. Tissues were then mounted onto superfrost plus slides, allowed to air dry and baked at 50°C for 20 min. For quantification of myelin content and calbindin and IBA-1 immunoreactivity three tissue sections per mouse were stained and eight random images per sections were acquired. Individual cells were counted using the count analysis tool in Adobe Extended CS6v13 (Adobe) and immunoreactive area obtained in Image J as previously described (Mendiola et al., 2017).

### Flow Cytometry and Cell Sorting

Mononuclear cells were isolated from brain and spinal cord of CX3CR1-WT, CX3CR1-KO, and hCX3CR1^I249/M280^ mice by homogenization of CNS tissues as previously described (Pino and Cardona, 2011). Cellular suspensions were blocked using anti-mouse CD16/CD32 (clone 2.4G2, BD Pharmingen) followed by incubation for 30 min with a mix of fluorochrome-conjugated anti-mouse antibodies as follows. Antibody cocktail for mononuclear myeloid cells and activation markers: CD45 APC-Cy7 (clone 30-F11, BioLegend), CD11b PE-CF594 (clone M1/70, BD Biosciences), CD11c PE-Cy7 (clone N418, eBioscience), I-A/I-E BV421 (MHC-II clone M5/114.15.2, BD Biosciences), CD86 PerCP/Cy5.5 (clone GL-1, BioLegend), Ly6C AF647 (clone ER-MP20, Serotec), Ly6G PE (clone 1A8, BD Pharmingen). T cells were characterized using the following antibody mix: CD45 eFluor 450 (clone 30-F11, eBioscience), CD3e PE-Cy7 (clone 145-2C11, BioLegend, CD8a APC (clone 53-6.7, eBioscience), CD4 APC-Cy7 (clone RM4-5, BioLegend), CD44 PerCP (clone IM7, eBioscience). Samples were acquired on an LSRII (BD Biosciences) at the Cell Analysis Core, UTSA. Data analysis was performed using FlowJo v10. For cell sorting, brain cell suspensions (pooled 3 mice per sample) were stained with antibodies against CD45 and CD11b and after gating for single cells the CD45^Lo^CD11b^+^ population was separated in a FACS ARIA-II (BD Biosciences).

### Intracellular Staining and Imaging Flow Cytometry

To assess cytokine production on T cells infiltrating the CNS, immunized mice were sacrificed at peak disease and mononuclear cells from brain tissues were isolated as previously described (Garcia et al., 2013). T cells were stimulated for 4 h at 37°C and 5% CO_2_ under the following conditions: cRMPI [RPMI 1640, Gibco; 10% Fetal bovine serum, Atlanta Biologicals; 1% Pen-Strep (Gibco), anti-CD3e (BioLegend), anti-CD28 (BD Pharmingen) and GolgiStop (BD Biosciences)]. For intracellular cytokine staining, the following reagents were used: Mouse Fc block (clone 2.4G2, BD Pharmingen); fixation/permeabilization buffer (Invitrogen); perm/wash buffer (BD Biosciences); cell staining buffer (BioLegend) and the following antibody cocktail: TCR-b V450 (clone H57-597, BD Horizon), CD4-APC-Cy7 (clone RM4-5, BioLegend), CD8a-PerCP/Cy5.5 (clone 53-6.7, BD Pharmingen), IL-17-PE (clone TC11-18H10.1, BioLegend) and IFN-γ-APC (clone XMG1.2, BioLegend) Samples were acquired using an ImageStreamX-Imaging Flow Cytometer-ISX-MKII (EMD Millipore) at the Cell Analysis Core, UTSA. Data analysis was performed using IDEAS software version 6.2.

### Cytokine ELISpot Assay

Mice were immunized with 100 μg MOG_35-55_ and sacrificed 10 days post-immunization. Inguinal lymph nodes were harvested and cells were plated at a density of 5 × 10^5^ per well. Assay was performed as previously described (Garcia et al., 2013). Plate was read and spot counted using ImmunoSPOT software. Data are presented as spot-forming units per million of cells.

### Enzyme-Linked Immunosorbent Assay

For the detection of soluble FKN, IL-1β and ciliary neurotrophic factor (CNTF) in CNS tissues, mice were anesthetized with 5% isoflurane in an induction chamber, followed by perfusion with ice cold Hanks balanced salt solution (HBSS w/o Ca^++^, Mg^++^, Lonza). Spinal cord, cerebellum, and forebrain were dissected and disrupted manually using dounce homogenizers. Suspensions were made in lysis buffer containing 150 mM NaCl, 10 mM Tris-Base, 1 mM EDTA pH 8.0 and 1X protein inhibitor cocktail (Roche). Lysates were centrifuged at 12,000 rpm for 15 min at 4°C, aliquoted and stored at -80°C. Total protein concentrations were determined using the Bio-Rad protein reagent assay (Bio-Rad) and quantities of soluble factors were measured by enzyme-linked immunosorbent assay (ELISA) using the following kits: DuoSet ELISA development system (R&D Systems) for mouse CX3CL1/Fractalkine, DuoSet ELISA development system (R&D Systems) for mouse IL-1b/IL-1F2 and ELISA kit (CUSABIO) for the mouse CNTF. Samples were run in duplicate. Results were normalized to total protein and reported in picogram (pg) amounts per milligram (mg) of protein as previously described (Cardona et al., 2015).

### NanoString Gene Expression Analysis and RNA Sequencing

Total RNA (100 ng) from cerebellar extracts at chronic EAE was used to analyze gene expression changes using nCounter and nSolver 3.0 software (NanoString Technologies). Transcript levels of 582 genes were assessed with the nCounter GE-Mouse Immunology v1 Kit (NanoString Technologies) performed by the Genomic and RNA Profiling Core Baylor College of Medicine (Department of Advanced Technology Cores). Data were initially normalized using positive and negative controls and housekeeping specific probes (Mendiola et al., 2017). Samples were then further normalized within nSolver to naïve groups. Differentially expressed genes were defined by a cutoff of ±2-fold change and *p* < 0.05. For RNA sequencing, sorted cells were subjected to total RNA extraction using the PicoPure RNA isolation kit (Arcturus), libraries were obtained from the genome sequencing facility (Greehey Children’s Cancer Research Institute – UT Health San Antonio). Raw reads were imported into CLC Genomics Workbench v10.1.1. Initially, low quality reads were trimmed; remaining reads for each sample were mapped to the annotated mouse genome (GRCm38), followed by differential gene expression (DGE) analysis of EAE samples from CX3CR1-WT, CX3CR1-KO, and hCX3CR1^I249/M280^ mice against their respective naïve controls using the RNA-seq tool within CLC Genomics Workbench software. Genes were deemed to be differentially expressed if the false discovery rate (FDR) was 0.05 or less.

### Computational Methodology and Molecular Modeling of CX3CR1 Protein

Search for CX3CR1 in the Protein databank RCSB (Berman et al., 2012) provided information for the crystallized form of CCR5 identified as 4MBs (Tan et al., 2013) that was used as template to predict the structure of CX3CR1 constructing hypothetical models. The primary sequence of *Homo sapiens* CX3CR1 is reported in the Uniprot database in FASTA format and was used for structural prediction model (UniProt Consortium, 2012, 2014). The hypothetical structural model for CX3CR1 was obtained using the peptide and protein structure predictor Phyre2 (Kelley and Sternberg, 2009) which belongs to the group of structural bioinformatics at the Imperial College of London, and allows an assembly by homology based on comparison of the reported crystalized structure and primary sequences. The predictor software includes an algorithm that favors the identification of structural folds and via threading methodology, assembles fragments found by similarity utilizing the atomic backbone as a flexible system that follows the dynamic laws of Newton in classical conceptualization (Kelley et al., 2015). To characterize and evaluate the obtained hypothetical models, a validation was performed using a tool from the group of bioinformatics and crystallography RAMPAGE [8], that takes into account the special distribution and orientation, calculating in graph of quadrants, Phi (φ) and Psi (ψ) dihedral angles of the peptide bonds that join each amino acid in the model. Likewise, an energetic validation is performed from the suite Swiss assessment structure as QMEAN6 (Benkert et al., 2008; Studer et al., 2014) and Z-score with the PROSA web software (Wiederstein and Sippl, 2007) that via comparative methodology classifies the models based on experimentally obtained and characterized structures, and the software then assigns a score to select the best model for a size of 355 amino acids, corresponding to size of CX3CR1 (Sippl, 1993). Structural viewers were used to gain further insights into the special and tridimensional distribution of the receptor. Deepview/Swiss-PdbViewer v3 (Guex and Peitsch, 1997) allowed us to do a preliminary assessment and then structures were visualized in the UCSF Chimera v1.11 (Pettersen et al., 2004) as it allows configuration of the model as ribbon, surface, or density type based on the spatial volume.

### Bioinformatics Analysis of CX3CR1 Variant

Native CX3CR1-WT (V249/T280) and variant CX3CR1^I249/M280^ proteins were analyzed for hydropathic index, N-glycosylation, and quantification of the transmembrane domain with the hidden models of Markov. This procedure uses the primary protein sequence in FASTA format and by selecting the Protscale software that belongs to the Swiss ExPASY suite, the menu allows the calculation of the hydropathic index selecting the Kyte and Dolittle coefficients (Artimo et al., 2012) which assigns a score to each amino acid. The index ranges from positive to negative numbers that is equivalent to hydrophobic and hydrophilic values respectively, with zero as the separation baseline. For characterization of N-glycosylation of the protein we use NetGlyc 1.0 server which calculates the probability of N-glycosylation in the sequence of amino acids Asn-Xaa-Ser/Thr (Artimo et al., 2012). Since CX3CR1 is a seven-transmembrane domain receptor, we performed an assessment of the transmembrane domains with the TMHMM 2.0 Suite Swiss-ExPASy, to locate the amino acids within the membrane and intracellular-extracellular loops with scores obtained with “websequence” (Artimo et al., 2012). The theoretical orientation and positioning of the protein within the lipid bilayer is assembled with the software web PPM server that takes into account hydrophobic interacting forces, distances and interaction of hydrogen bonds with the aqueous environment, bonding profiles due to the dielectric constant of water, dipolar interaction with protein residues and the anisotropy of water that, based on an stochastic focus, provides a model to define the topology of the system (Lomize et al., 2012).

### Statistical Analysis

Data are expressed as mean ± SEM in graphs and plots. In scattered plots, each dot represents data from an individual mouse. Data were plotted in GraphPad Prism 5 and statistical comparisons performed by non-paired two-tail Student’s *t*-test or One-way ANOVA followed by Tukey’s post-test when comparing multiple groups. *p*-Values < 0.05 considered statistically significant. RNA seq data was subjected to exact test comparison of EAE tissues against their naïve counterparts. Bonferroni and FDR corrections were applied. FDR *p*-values < 0.05 were considered statistically significant.

## Results

### Defective CX3CR1 Signaling Through hCX3CR1^I249/M280^ Variant Exacerbates Clinical EAE Disease

**FIGURE 1:**
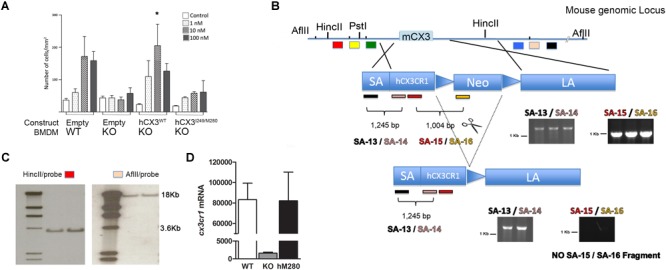
Generation of mice expressing the human CX3CR1^I249/M280^ variant. **(A)** To confirm that human CX3CR1 signals effectively in response to mouse FKN, CX3CR1-KO bone marrow derived macrophages were transfected with pIRES expression vectors expressing the respective human sequences WT (V249/T280), or M280 (I249/M280) CX3CR1. Cells were compared in migration assays in response to various concentrations of soluble recombinant mouse fractalkine. Transfection of vectors into KO or WT macrophages show dose dependent transmigration of cells in response to mouse recombinant FKN. Samples were run in duplicates and data represents the average of three independent experiments. **(B)** Targeting construct containing the hCX3CR1^I249/M280^ sequence was engineered using the pEasyflox vector harboring a 12 Kb fragment containing the 5′ mouse CX3CR1 UTR (Short Arm, SA) upstream of the human CX3CR1 start codon, and a 7.0 Kb fragment spanning the 3′ mouse UTR [long arm (LA)], and targeting strategy depicting the location of probes for validation by Southern blots, and PCR genotyping. **(C)** After delivery of the targeting constructs to ES cells, positive clones with successful homologous recombination were identified by Southern blots upon HincII and AflII digestion to confirm insertion at both 5′ and 3′ ends with SA and LA specific probes. **(D)** Expression of CX3CR1 was confirmed at the transcript levels in sorted microglial cells from corresponding, CX3CR1-WT, KO, and hCX3CR1^I249/M80^ *n* = 3 samples per group. ^∗^*p* < 0.05.

**FIGURE 2:**
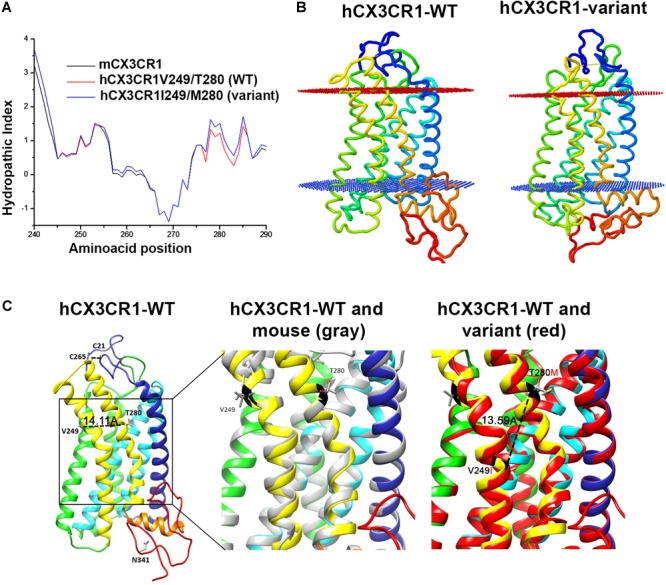
CX3CR1 hypothetical tridimensional model. **(A)** The hydropathic index provides information on the effects of amino acid changes to the chemical environment of the protein. The index based on the coefficients of Kyte and Dolittle show that there is an increase in the hydrophobicity in the region with the change T280M. **(B)** The orientation and position of the protein within the lipid bilayer is assembled with software web PPM and **(C)** the aligned hypothetical models of the human-reference receptor and mouse CX3CR1 indicate that the protein structure and residues 249/280 align in a similar conformation (dashed line inside box) 14.11 Å apart, whereas in the variant receptor, I249/M280 residues fall closer within the structure 13.59 Å.

**FIGURE 3:**
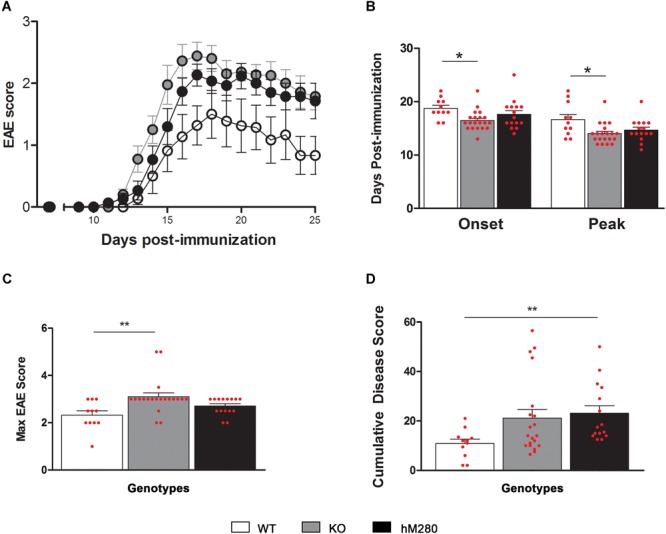
hCX3CR1^I249/M280^ expressing mice and CX3CR1-KO mice exhibit higher EAE scores. **(A)** CX3CR1-WT (open circles/bars) CX3CR1-KO (gray circles/bars) and hCX3CR1^I249/M280^ mice (black circles/bars) were immunized with MOG_35-55_ peptide and scored daily for neurological signs. **(B)** Days of EAE onset and peak, **(C)** maximum EAE score, and **(D)** cumulative disease score. ^∗^*p* < 0.05, ^∗∗^*p* < 0.01. Each dot represents data from an individual mouse.

Data from our laboratory has shown that fractalkine/CX3CR1 signaling regulates microglia neurotoxicity in selected mouse models of neurodegeneration (Cardona et al., 2006; Bhaskar et al., 2010; Garcia et al., 2013; Maphis et al., 2015). To complement those studies our laboratory has developed a new animal model that allows the understanding of not only CX3CR1/FKN during models of neuroinflammation, but to uniquely define the role of the human CX3CR1 variant during MS models. For this, we engineered a transgenic mouse in which the human CX3CR1 variant gene (hCX3CR1^I249/M280^) was introduced into the mouse genome replacing the mouse counterpart (**Figure 1**). Comparison of protein sequence between the WT and variant receptors revealed that the T280M substitution contributes to an increase in the hydrophobicity of the region between amino acids 275 and 290 (**Figure 2A**). The hydropathic index is important to understand how the mutations in the variant receptor affect the protein regions potentially involved in the interaction ligand–receptor. A tridimensional model of the variant receptor proposes an alteration in the interaction with the cellular membrane resulting in a shift toward the intracellular space of the alpha-helix containing the V249I substitution (**Figures 2B,C**). This shift has a significant effect in the extracellular portion of the protein as it results in the disruption of disulfide bridges (C21–C265), thereby generating conformational changes in the protein structure that alter CX3CR1-FKN recognition domain which could potentially negatively affect protein function (**Supplementary Figure S1**). Results indicate that after active immunization with MOG_35-55_ peptide, clinical disease was more severe in mice expressing the human CX3CR1 variant when compared to WT mice (**Figure 3A**). Interestingly, the time at which the hCX3CR1^I249/M280^ mice reached their highest clinical disability (**Figure 3B**, peak) resembled the observations of the CX3CR1-KO mice. Despite the fact that the maximum score observed in the hCX3CR1^I249/M280^ mice showed an intermediate value between the WT and CX3CR1-KO mice (**Figure 3C**), the cumulative disease score, a measure of clinical disease severity closely resembled and reproduced previous observations from our laboratory reporting an exacerbated phenotype in mice lacking CX3CR1 (**Figure 3D**). These data support the notion of impaired fractalkine signaling as a result of the expression of the adhesive deficient human I249/M280 polymorphic variant.

### Expression of hCX3CR1^I249/M280^ Is Associated With Severe Cerebellar Inflammation and Neuronal Pathology

**FIGURE 4:**
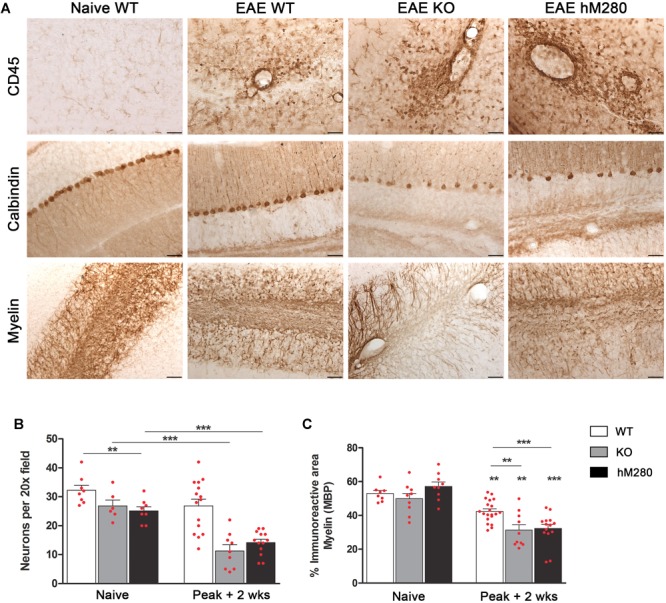
CX3CR1^I249/M280^ and CX3CR1-KO mice reveal increased cerebellar pathology. **(A)** Brain tissues were subjected to immunohistochemistry with antibodies against CD45 (brown staining) as a marker of global inflammation (top panels), calbindin to visualize cerebellar Purkinje cells (middle panels) and MBP to compare demyelination in WT, KO, and hCX3CR1^I249/M280^ mice (hM280). Scale bars 50 μm, original magnification 20x. **(B)** Calbindin-positive cells per section, ^∗∗^*p* < 0.01 between naïve WT and hM280 mice and ^∗∗∗^*p* < 0.001 between diseased KO and hM280 compared to WT mice. **(C)** Myelin immunoreactive area, *p <* 0.01 when comparing EAE to corresponding naïve mice, and across the selected groups, ^∗∗∗^*p* < 0.001.

To investigate the association between clinical manifestation and CNS pathology, we assessed infiltration and neuronal damage in brain sections of mice sacrificed at the chronic phase of EAE. Higher clinical scores during EAE were associated with severe infiltration of blood-derived inflammatory cells to the CNS and their actions on axonal fibers that lead to the ataxia and paraplegia observed in the time course of the disease. Persistent extravasation of inflammatory cells, particularly into the cerebellum of immunized mice, are depicted by the increase in CD45 staining upon EAE induction (**Figure 4A**, top). Severe inflammation in mice expressing the hCX3CR1^I249/M280^ receptor correlated with significant decrease in calbindin^+^ cells in the cerebellum (**Figures 4A** middle,**B**) based on the quantification of neurons in the Purkinje cell layer and its comparison to immunized WT mice. Neuronal loss in CX3CR1-KO and hCX3CR1^I249/M280^ mice correlated with worsened demyelination identified by the loss of MBP staining in the cerebellar white matter when compared with myelin staining in naïve mice (**Figures 4A** bottom,**C**). These observations recapitulate the cerebellar pathology observed in the CX3CR1-KO mice, suggesting a crucial role for defective CX3CR1 signaling in the inflammatory mediated events that lead to the development of pathological features during MOG_35-55_ induced neurodegeneration.

### Inflammatory Response in CNS of hCX3CR1^I249/M280^ Mice Is Dominated by Macrophages and T Cells at Peak EAE

**FIGURE 5:**
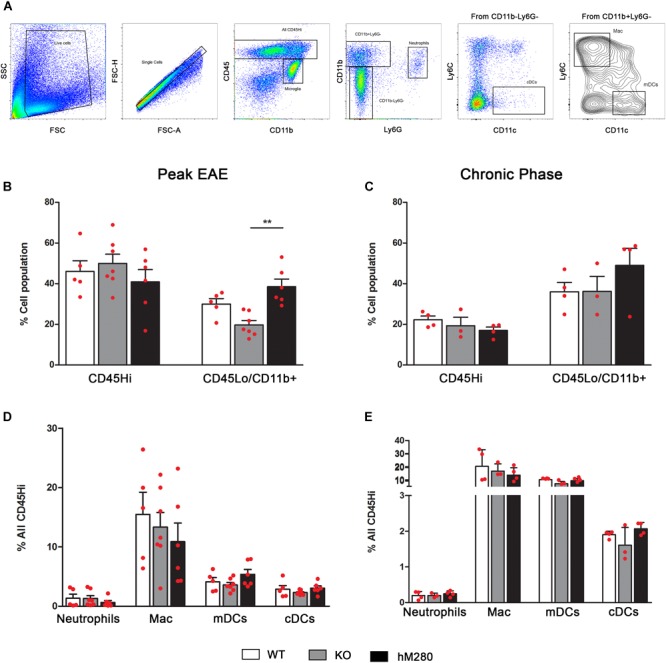
Predominant abundance of CD45^Lo^CD11b^+^ cells in hCX3CR1^I249/M280^ mice at peak phase of disease. **(A)** Gating strategy of brain mononuclear cells separated over Percoll gradients at peak and chronic stage of disease. Cells were stained and analyzed by flow cytometry, singlets were gated and cells separated to distinguish microglia (CD45^Lo^CD11b^+^), neutrophils (CD45^Hi^CD11b^+^Ly6G^+^), macrophages (CD45^Hi^CD11b^+^Ly6C^Hi^Ly6G^-^), monocyte derived DCs (CD45^Hi^CD11b^+^Ly6G^-^CD11c^+^) and conventional DCs (CD45^Hi^CD11b^-^Ly6G^-^CD11c^+^). **(B–E)** Frequency of total infiltrating cells, microglia, and myeloid subsets at peak and chronic stage of disease are shown, each point represents data from an individual mouse. ^∗∗^*p* < 0.01.

**FIGURE 6:**
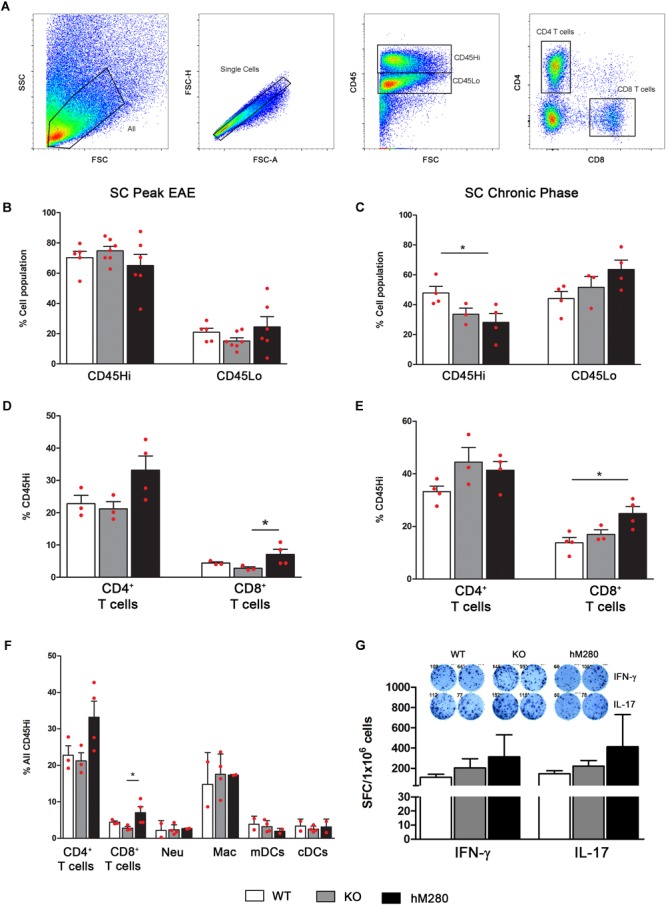
Increased abundance of CD8 T cells in spinal cord tissues of hCX3CR1^I249/M280^ mice. **(A)** Gating strategy of spinal cord cell suspensions separated over Percoll gradients at peak and chronic stages of disease. Cells were stained and analyzed by flow cytometry, singlets were gated and cells separated to distinguish CD45^Hi^ blood derived inflammatory cells and CD45^Lo^ microglia at peak **(B)** and chronic stages of disease **(C)**. CD4^+^ and CD8^+^ T cells as CD45^Hi^CD4^+^CD8^-^ and CD45^Hi^CD4^-^CD8^+^ respectively were also compared at peak **(D)** and chronic **(E)** stages of EAE. A separate staining cocktail as described in the data presented in **Figure 5** for myeloid subsets was included and frequency of lymphocytes and myeloid populations at peak disease compared **(F)**. **(G)** Cytokine ELISpot assay to compare the frequency of IFN-γ and IL-17 producing T cells that are specific for MOG_35-55_ peptide is shown. ^∗^*p* < 0.05. Each point represents data from an individual mouse.

Analysis of the inflammatory response in EAE affected brains by flow cytometry analysis (**Figure 5A**) reveals a higher proportion of CD45^Hi^ cells at peak disease (**Figure 5B**) compared to chronic stages when CD45^Lo^/CD11b^+^ microglia appear as the predominant cell population (**Figure 5C**). Interestingly, the proportion of microglia at peak EAE in hCX3CR1^I249/M280^ mice slightly exceeded what was observed in CX3CR1-KO mice (**Figure 5B**). Assessment of myeloid populations revealed CD45^Hi^CD11b^+^Ly6C^+^Ly6G^-^CD11c^-^ macrophages (**Figure 5A**) as the most abundant myeloid cells in the brain of EAE-induced mice both at peak and chronic EAE (**Figures 5D,E**), regardless of the CX3CR1 genotype. Also, comparable levels of CD45^Hi^CD11b^+^Ly6G^-^Ly6C^-^CD11c^+^ myeloid derived dendritic cells (mDCs) (**Figure 5A**) were observed during chronic EAE (**Figure 5E**) across all genotypes. Flow cytometry analyses were also conducted in spinal cord tissues to classify the components of the inflammatory response in hCX3CR1^I249/M280^ mice (**Figure 6A**). Severe infiltration, reflected by the close to 70% of the total CD45^Hi^ population, was observed in the spinal cord of mice at peak disease (**Figure 6B**). Similarly to our observations in the brain, the severe inflammatory reaction appears to be the result of macrophages mobilization (**Figure 6F**), and excessive extravasation of CD4^+^ T cells (**Figures 6D,F**). Flow cytometry data from brains of EAE mice at chronic disease (**Figure 5C**) show a diminished inflammatory state by a reduction on the overall CD45^Hi^ population across all the genotypes. In contrast, WT mice exhibit a persistent state of cellular infiltration into the spinal cord when compared to hCX3CR1^I249/M280^ mice (and to CX3CR1-KO to a lesser degree) (**Figure 6C**). Previous studies revealed that autoreactive T cells against myelin proteins play a critical role on disease development during EAE. More specifically, T cell responses that result in the generation of IFN-γ secreting Th_1_ and IL-17 secreting Th_17_ CD4^+^ cells are involved in inflammatory-mediated damage to myelin and neurons in EAE. The activation of CD4^+^ T cells is initiated in peripheral lymph nodes with the binding to the antigenic peptide on major histocompatibility complex (MHC) class II and co-stimulatory molecules on antigen presenting cells (Wlodarczyk et al., 2014). Given that CX3CR1 expression is not restricted to CNS microglia, but is also confined to other subsets of circulating immune cells, we sought to evaluate the effect of the human CX3CR1 variant in the initiation of immune response against the MOG peptide during EAE. T cells were isolated from lymph nodes of actively immunized mice 10 days post-MOG_35-55_ immunization, re-stimulated *in vitro* and assessed for their polarization toward Th_1_/IFN-γ and Th_17_/IL-17 cells using the cytokine ELISpot immune assay (**Figure 6G**). Cytokine-producing T cells specific for MOG_35-55_ antigen were quantified as spot forming units and compared among WT, CX3CR1-KO and hCX3CR1^I249/M280^ genotypes (**Figure 6G**, top). Peripheral deficiency or defective CX3CR1-signaling did not have a negative effect in the priming and generation of pathogenic CD4^+^ T cells. Similar to the ELISpot results, IFN-γ and IL-17 producing CD4^+^ T cells were detected in the CNS (combined brain and spinal cord tissues) of mice at peak disease (**Supplementary Figures S2A,B**). These cells were harvested and, upon activation *in vitro*, showed no evident difference in cytokine production due to CX3CR1 signaling, supporting the notion that differential manifestation of EAE symptoms and CNS pathology does not seem to be the result of altered T cells responses in CX3CR1-KO or hCX3CR1^I249/M280^ mice. The abundance of T cells in the spinal cord of EAE mice was consistent with the notion that CD4^+^ T cells are the primary drivers of pathological alteration in this murine model (**Figures 6D,E**). Pathological data from MS patients shows that CD8^+^ T cells may play an important role in the propagation of the inflammation and tissue damage (Lassmann and Bradl, 2017). In this regard, it is of major interest that expression of the human variant CX3CR1^I249/M280^ receptor in the mouse genome was correlated with a higher percentage of CD8^+^ T cells both at peak and chronic EAE (**Figures 6D,E**) providing a relevant foundational argument for the utilization of these mice as a valid model for human inflammatory diseases of the CNS.

### Differential Transcriptional Profile in the CNS of EAE Induced Mice

**FIGURE 7:**
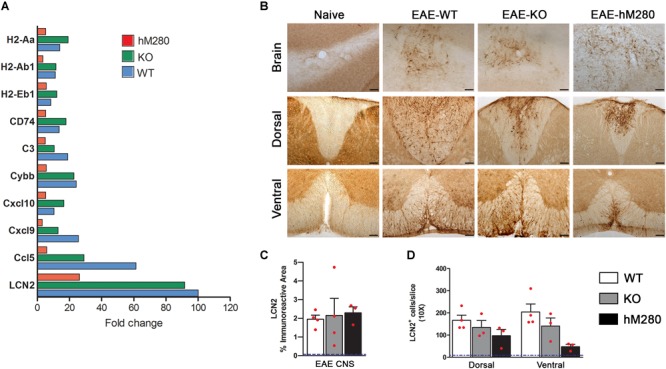
Nanostring gene expression analysis in the cerebellum of EAE induced mice. **(A)** Quantitative nCounter (Nanostring technologies) expression proofing of 582 immunological-related genes was performed on cerebellar tissues at chronic disease stages from CX3CR1-WT (blue-filled bars), CX3CR1-KO (green-filled bars) and hCX3CR1^I249/M280^ mice (red-filled bars). Top upregulated genes are shown. **(B)** To validate the expression of the top regulated transcript LCN2, immunohistochemistry was performed. Scale bars 100 μm, original magnification 10x. LCN2 immunoreactive area in brain **(C)** and cell density in spinal cord tissues **(D)** were compared among the groups. Each point represents data from an individual mouse. Dotted blue line represents the basal levels of LCN2 positivity in naïve tissues.

A fundamental concept underlying MS and EAE pathology is the associated inflammatory reaction, and the interplay between hematogenous derived cells and resident brain cells that contribute directly and/or indirectly to demyelination and axonal damage. In our efforts to further assess differences and similarities between the response to EAE in WT, CX3CR1-KO and hCX3CR1^M280^ mice, we performed NanoString analyses in which 549 transcripts within the immunology panel were compared. Due to the enhanced demyelination and neuronal pathology observed in cerebellar tissues of mice with defective CX3CR1 signaling, we focused this analysis in cerebellar regions among the EAE groups after normalization to naïve tissues (**Figure 7A**). LCN2 was the most upregulated gene in hCX3CR1^M280^ mice displaying a 26.19 fold increase, accompanied by higher levels in CX3CR1-KO mice (↑91.61) and WT mice (↑100.64). Increased LCN2 mRNA was validated at the protein level by immune-detection of LCN2 protein in brain and spinal cord tissues of EAE mice (**Figure 7B**). The immunopositive area was quantified in brain tisues (**Figure 7C**) and LCN2^+^ cells were counted in spinal cord (**Figure 7D**). LCN2 levels in naïve tissues are represented in dotted blue line in respective graphs (**Figures 7C,D**). Other major upregulated genes include Ccl5, Cxcl9, Cxcl10, cytochrome b beta (Cybb), complement component C3 (C3), and CD74. Inflammatory related genes upregulated in KO mice and with lesser abundance in hCX3CR1^M280^ mice include H2-Aa, H2-Ab1, and H2-Eb1.

### Microglia Responses During EAE

**FIGURE 8:**
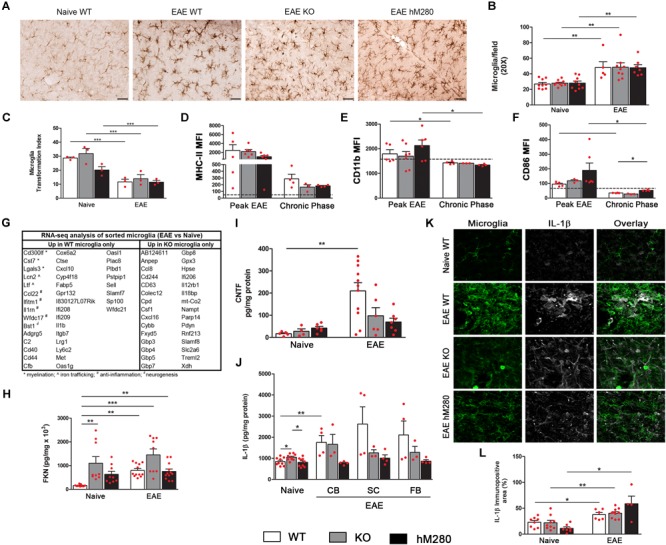
Microglia and effects of CX3CR1/FKN signaling during EAE. **(A)** Microglia cells were detected by immunohistochemistry staining using anti-Iba-1 antibody in brain tissues of EAE mice and compared to naïve counterparts. Scale bars 50 μm, original magnification 20x. Microglia were counted in cerebellum sections, ^∗∗^*p* < 0.01 **(B)** and morphological changes were measured by quantification of transformation index, ^∗∗∗^*p* < 0.001 **(C)**. Activation state of microglia (CD45^Lo^CD11b^+^) was also determined by flow cytometry assessment of median fluorescence intensity (MFI) of MHC-II **(D)**, CD11b **(E)** and CD86 **(F)**, baseline MFI of microglia in naïve tissues is represented by dotted line **(D–F)**. Microglia sorted based on CD45^Lo^CD11b^+^ profile were analyzed through RNA-seq. Table **(G)** shows a list of genes significantly upregulated only in CX3CR1-WT or CX3CR1-KO microglia as compared to naïve counter parts. Brain soluble extracts were used to measure FKN **(H)**, CNTF **(I)**, and IL-1β **(J)** levels in naïve and diseased mice. IL-1β expression was validated by immunofluorescence staining of cerebellar tissues of naïve and EAE mice **(K,L)**. ^∗^*p* < 0.05, ^∗∗^*p* < 0.01, and ^∗∗∗^*p* < 0.001.

Our laboratory and others have shown that FKN regulates microglia function by inhibiting microglia activation and the release of inflammatory mediators such as IL-1β. Induction of EAE results in increased microglia numbers in cerebellar tissues (**Figures 8A,B**) accompanied by changes in cell morphology, reflected by retraction of cellular processes and enlargement of the cell body (**Figure 8A**). Microglia activation is calculated by measurements of the roundness of individual cells as depicted by the transformation index (**Figure 8C**). Assessment of activation markers by microglia show an increased expression of MHC-II molecules during peak EAE, which were found ameliorated during chronic stages of the disease (**Figure 8D**). Negligible changes in the median fluorescence intensity (MFI) for CD11b (**Figure 8E**) or CD86 (**Figure 8F**) were observed in microglia in diseased mice, suggesting that antigen presentation through MHC-II is one of the most prominent features of microglia during EAE. Basal level for activation markers on naïve microglia are noted in dotted lines (**Figures 8D–F**). Infiltrating monocytes (CD45^Hi^CD11b^+^Ly6C^+^) showed increased MFI for MHC-II, CD11b, and CD86 only during peak EAE, with values comparable to naïve microglia by later stages of the disease. Activation markers on naïve blood macrophages did not show statistical differences across the genotypes (**Supplementary Figure S3**). Further RNA-seq analysis from microglia sorted from WT and CX3CR1-KO mice provided evidence for a role of microglia in myelination, iron trafficking, anti-inflammation, and neurogenesis driven by the expression of CX3CR1 (**Figure 8G**) as noted by the upregulation of genes involved in these physiological process only in WT microglia. In contrast, microglia from mice lacking the CX3CR1 receptor show upregulation of genes involved in inflammatory processes. Fold change and FDR *p*-value for significant genes (upregulated and downregulated) in CX3CR1-WT and CX3CR1-KO microglia during EAE are found in **Supplementary Tables S1, S2**.

### Deficient CX3CR1 Signaling Impairs Fractalkine and CNTF Production During EAE

Within the CNS, fractalkine has been proposed to act through the interaction with its unique CX3CR1 receptor expressed on microglia to maintain their homeostatic resting state. We found that basal fractalkine levels detected in soluble brain and spinal cord extracts of WT mice have a fivefold increase during EAE-induced disease (**Figure 8H**). A sevenfold increase in fractalkine was observed in naïve CX3CR1-KO mice in comparison to naïve WT mice, supporting a role for CX3CR1 as a scavenger receptor (Cardona et al., 2008). Fractalkine levels in CX3CR1 deficient mice seemed to reach maximum induction, as neuroinflammation induced by MOG_35-55_ immunization did not affected significantly the levels of detected soluble fractalkine. Similar results were observed in mice expressing the human CX3CR1^I249/M280^ variant, which exhibited intermediate fractalkine levels in naïve conditions that appear sustained upon EAE induction (**Figure 8H**). Similar to the effects on fractalkine production, we observed impaired production of CNTF in the CNS of both mouse strains with defective CX3CR1 signaling, CX3CR1-KO and hCX3CR1^I249/M280^ mice at chronic EAE (**Figure 8I**). Also, the defect on CNTF production correlated with the significant neuronal cell loss observed as a result of CX3CR1 dysfunction in CX3CR1-KO and hCX3CR1^I249/M280^ mice. To address if IL-1β levels were differentially regulated in the diseased brain, cytokine ELISA was performed (**Figure 8J**), and combined with IL-1β immunostaining, the data indicate that soluble and cell-associated IL-1β is increased more abundantly in WT mice (**Figures 8J–L**).

## Discussion

Chemokines and their receptors play an important role during CNS autoimmune inflammation, not only by guiding immune cell infiltration within demyelinating lesions but also by promoting a communication system for resident CNS cells. As an example, FKN and CX3CR1 are abundantly expressed in the naïve CNS by neurons and microglia respectively. FKN and CX3CR1 play an important role in brain homeostasis and the severe phenotype of mice lacking FKN or CX3CR1 in selective models of neurodegeneration poses a question about the impact of polymorphic CX3CR1 variants during neuroinflammatory insults. In this study, we describe the phenotype of mice expressing the human CX3CR1^I249/M280^ variant during active EAE. This novel model shared phenotypic features seen in EAE-induced CX3CR1-WT and CX3CR1-KO mice. Notable findings are highlighted by comparable EAE severity and cerebellar pathology in hCX3CR1^I249/M280^ and CX3CR1-KO mice (**Figure 4**), and abundance of microglia and CD8^+^ T cells in hCX3CR1^I249/M280^ mice (**Figure 6**). Although this report does not include a direct comparison to the reference hCX3CR1^V 249/TM280^, this humanized system serves as a proof of principle to validate alternative interventions/therapies to modulate the inflammatory reaction in the EAE brain with direct translational relevance to human disease and will provide valuable information on the function of these polymorphic receptors in various immune cell types.

We showed *in vitro* that the reference human receptor was able to signal in response to mouse FKN and induce cell migration in a dose dependent manner, whereas the variant hCX3CR1^I249/M280^ displayed blunted responses, correlating with published studies that established the adhesive defective properties of the hCX3CR1^I249/M280^ (McDermott et al., 2003). Protein bioinformatic and hypothetical modeling revealed that the amino acid changes in the variant receptor affect the hydrophobicity of the protein within the intermembrane region. The structure within the intramembrane domain did not appear to differ between the mouse CX3CR1-receptor and the hCX3CR1^V 249/T280^ reference receptor. However, for the variant hCX3CR1^I249/M280^ receptor, these changes also affect the extracellular loops that contact the ligand. Functionally, the model was validated by (1) assessing chemotactic responses toward the mouse FKN in transmigration assays, (2) confirming homologous recombination of the target construct by Southern blots using 3′ and 5′ probes, and (3) comparing CX3CR1 expression at the transcript level in CNS tissues (**Figure 1**) across the genotypes, showing that mice expressing the hCX3CR1^I249/M280^ variant receptor exhibit CX3CR1 mRNA levels comparable to WT mice.

Analyses of cellular infiltration revealed comparable frequencies of IFN-γ and IL-17 MOG_35-55_ antigen specific T cells in draining lymph nodes. Similarly, myeloid cell infiltration and abundance of CD4^+^ T cells did not reveal significant differences among CX3CR1-WT, CX3CR1-KO, and hCX3CR1^I249/M280^ groups. However, CD8^+^ T cell infiltration appeared to be increased in mice expressing the variant receptor. Similarly, microglial frequency quantified by flow cytometry appear increased in hCX3CR1^I249/M280^ mice. Of interest was the distinct transcriptional profile of WT versus KO CNS tissues during the chronic phase of EAE. Using the inflammatory code set from nanostring technologies, lipocalin 2 (LCN2) was the top upregulated gene with a higher abundance in both CX3CR1-WT and CX3CR1-KO tissues compared to hCX3CR1^I249/M280^. Validation at the protein level revealed a similar pattern of expression in brain tissues in agreement with other EAE studies (Marques et al., 2012). LCN2 is a multi-functional protein that plays a role in cell migration (Lee et al., 2011), inflammation (Nam et al., 2014), neurogenesis (Ferreira et al., 2018), iron transport (Srinivasan et al., 2012), and reduction of oxidative stress (Yamada et al., 2016). The phenotype of LCN2-KO mice during EAE has produced conflicting results with one study reporting ameliorated disease after EAE induction (Nam et al., 2014) and another study showing inhibition of remyelination by LCN2 *in vitro* (Al Nimer et al., 2016). The data presented here suggest that less severe EAE in WT mice correlates with higher LCN2 levels. Since iron has been proven essential for progenitor cell proliferation and differentiation (Schonberg and McTigue, 2009), understanding how FKN directly or indirectly regulates LCN2 expression in glial cells may provide valuable insights on ways to enhance neuroprotection. Following up the overall tissue transcriptional profile, RNA sequencing of sorted microglia (**Figure 8**) also revealed several significant differentially regulated genes, with particular relevance in WT microglia transcripts involved in myelination (Cd300lf, Lgals3, Cst7), iron trafficking (LCN2, Ltf), neurogenesis (Bst1), and anti-inflammatory pathways (Ccl22, Ifitm1, IL1rn). Due to the expression of IL-1 related transcripts and the association of IL-1β with microglial neurotoxicity (Cardona et al., 2006) we assessed protein expression by staining and tissue ELISA and found that soluble IL-1β is higher in WT tissues with expression likely restricted to infiltrating immune cells, whereas in CX3CR1-KO and hCX3CR1^I249/M280^ mice most IL-1β^+^ cells colocalized with GFAP^+^ staining. Also, of relevance is the observation of decreased CNTF protein levels in CX3CR1-KO and CX3CR1^I249/M280^ mice, in particular because CNTF is widely recognized as a key survival factor not only for neurons but also for oligodendrocytes (Stankoff et al., 2002; Kuhlmann et al., 2006; Tebar et al., 2008; Cao et al., 2010). Similar to LCN2, the mechanisms by which CX3CR1 signaling promote CNTF production are still to be dissected. Future studies comparing the transcriptome profile of microglia expressing the reference and variant receptors would be valuable to better understand the regulation of microglia by CX3CR1 within the human population.

The combined actions of genetic and environmental factors influence susceptibility to autoimmune disease (Reich et al., 2018). In the context of MS, close to 200 gene variants that increase the risk of MS have been identified in genome wide association studies of which the most significant remains the MHC (Sawcer et al., 2011). Other risk alleles include inflammation-related mediators. The biology of FKN/CX3CR1 presents various aspects of relevance to the pathology of MS, including the CX3CR1 and FKN expression pattern. CX3CR1 is expressed by microglia and subpopulations of circulating monocytes, dendritic cells, T cells, and NK cells. However, microglia, relative to hematogenous derived cells, express the highest levels of CX3CR1 (Saederup et al., 2010; Mizutani et al., 2012). Increased FKN levels are present in cerebrospinal fluid and serum of patients with relapsing-remitting MS (Kastenbauer et al., 2003) and comparison of MS and healthy post-mortem brains revealed the presence of a hypermethylation mark in the FKN loci that correlated with decreased FKN transcript levels in demyelinated lesions (Huynh et al., 2014). In a recent study, it was suggested that the hCX3CR1-M280 mutation associates with secondary progressive MS (SPMS) in which patients develop more demyelinated lesions (Stojkovic et al., 2012). Moreover, CX3CR1 mRNA is present in active MS lesions, although it is unknown which polymorphic variant was present (Ridderstad et al., 2014). In a rat EAE model, subcutaneous administration of high doses of a chemokine inhibitor ameliorated disease symptoms. However, specificity toward CX3CR1 versus other chemokine receptors was not defined. Moreover, this study did not provide characterization of microglial responses, nor CNS immune subsets to determine if the effects of the inhibitor were due to influences in peripheral or CNS resident cells (Ridderstad et al., 2014). CX3CR1^+^CD4^+^ T cells were enriched in cerebrospinal fluid of relapsing-remitting MS patients (Blauth et al., 2015) and CX3CR1 appears critical for the development of CD8 memory T cells (Bottcher et al., 2015). It was also found that CX3CR1 recruits regulatory NK cells and CX3CR1-deficiency exacerbates active EAE disease (Huang et al., 2006). MS patients revealed lower expression of CX3CR1^Hi^ NK cells in peripheral blood which correlated with disease activity (Infante-Duarte et al., 2005), and CX3CR1 was required for maturation of NK cells and a protective role of CX3CR1^Hi^NK cells via elimination of autoreactive T cells is proposed (Hamann et al., 2011; Hertwig et al., 2016). EAE-induced CX3CR1-KO mice revealed increased infiltration of monocyte derived DCs to the brain, greater microglia activation and proliferation associated to areas of enhanced demyelination and neuronal loss (Garcia et al., 2013). Overall, EAE phenotype in mice expressing the hCX3CR1^I249/M280^ variant show higher disease scores, significant demyelination, decreased cerebellar neuronal counts, higher CD45^Lo^CD11b^+^, and CD8 T cell densities. Using nanostring analyses from the top significantly upregulated genes among WT, KO and hCX3CR1^I249/M280^ mice, LCN2, Ccl5, Cxcl9 and genes related to MHC-II antigen processing and presentation were in lesser abundance in hCX3CR1^I249/M280^ mice. Previously published observations support the relevance of FKN/CX3CR1 pathway in CNS pathology, the results in the current study establish the potential risk of worsening disease severity in a hCX3CR1^I249/M280^ expressing host. Future studies guided to compare microglia, and CD8 T cells responses in mice alternatives models engineered to express the human reference and variant CX3CR1 receptors will be critical to validate the data presented here and will overall provide a better understanding of the impact of this polymorphic variants in optic nerve pathology and models of demyelinating disease.

## Author Contributions

SC designed experiments, performed research, analyzed data, and wrote the article. SK performed research, analyzed data, and revised the manuscript critically. VT, KC, IC, SS, and AM performed research. SW revised the work critically and provided intellectual resources. JP-T performed research and revised the work critically. AO and PA analyzed and interpreted data. RR conceived the study. AC conceived the study, designed experiments, performed research, analyzed data, and wrote the article.

## Conflict of Interest Statement

The authors declare that the research was conducted in the absence of any commercial or financial relationships that could be construed as a potential conflict of interest.

## Supplementary Material

FIGURE S1 Quantitative validation of energetics and stereochemistry. **(A)** The proposed structural models were built using the prediction software Phyre2, Swiss-assessment structure suite that provides energetic values and the RAMPAGE software that characterizes the stereochemistry of the proposed model from measurement of dihedral angles with Ramachandran graphics, showing that 89% of the residues fall within the favored region, 6.5% in the allowed region and 4.5% in the outliner region, therefore 95.5% of the residues are within the degree of acceptance for the QMEAN6/Z score and Ramachandran plot. **(B)** Comparison of protein composition based on size given by *Z*-score values. The feasibility of the hypothetical model yields a value of -3.81, positioning the model within the *X*-ray and NMR area of the graph based on experimental data. **(C)** Energetic characterization based on fragments of amino acids along the primary sequence, first using a central amino acid and obtaining an energetic average in fragments of 10 and 40 amino acids (window size). Values above the ‘zero’ threshold are considered problematic and coincide within extracellular and intracellular loops that reflect flexible and disorganized areas that may function as the recognition site of the ligand fractalkine. Similarly, the QMEAN6 value obtained is derived from a combination of linear values including the energy of torsion angles, coupling energy of atom pairs, energy of solvation, and energy of the beta carbon, values of secondary structure and values of accessibility of solvent. The model is assigned a normalized value that estimates the reliability of the model with a range between 0–1. For the selected model the QMEAN6 value of 0.546 as a global parameter that is accepted when values are above 0.5. **(D)** The most reliable amino acid fragments coincide with the regions that are not mobile such as α-helices within the membrane (**Figure 2**). Loops are predicted with a disorganized orientation but with an extracellular configuration that may be involved in ligand-receptor interaction. The variant protein proposes a shift that affects the extracellular region leading to disruption in disulfide bridges (C21-C265), generating conformational changes in the protein structure that alter CX3CR1-ligand recognition domain and which could potentially negatively affect protein function.

FIGURE S2 IL-17 and IFN-γ production by CD4^+^ and CD8^+^ T cells in EAE affected mice. **(A)** Brain and spinal cord mononuclear cells isolated over percoll gradients were subjected to imaging flow cytometry with gating strategy based on singlet population, followed by CD4 and CD8 T cell separation and IFN-γ and IL-17 discrimination. **(B)** Images show that double positive cells for IFN-γ/IL-17 were less abundant that the single positive counterparts and detected in similar proportions among the groups.

FIGURE S3 Signature profile of brain and blood leukocytes. Activation of brain and spinal cord microglia (CD45^Lo^CD11b^+^) and infiltrating macrophages (CD45^Hi^CD11b^+^Ly6C^+^) was assessed by flow cytometric assessment of median fluorescence intensity as a representation of abundancy of MHC-II (left panels), CD11b (middle panels) and CD86 (right panels) molecules. These activation markers were also evaluated in blood monocytes from naive mice. Microglia activated phenotype is characterized by increased MHC-II MFI at peak disease, whereas infiltrating monocytes also exhibit higher MFI for CD11b and co-stimulatory molecule CD86. Insertion of hCX3CR1^I249/M280^ does not appear to affect the peripheral immune populations (bottom panel). Presented *p*-values correspond to significant differences between mice of the same phenotype at peak vs. chronic disease, unless otherwise stated.

TABLE S1 Genes significantly regulated in CX3CR1-WT microglia in comparison to naïve microglia.

TABLE S2 Genes significantly regulated in CX3CR1-KO microglia in comparison to naïve microglia.
