# Supplementary figures and images for: Role of the Fractalkine Receptor in CNS Autoimmune Inflammation: New Approach Utilizing a Mouse Model Expressing the Human CX3CR1I249/M280 Variant
Source: Front Cell Neurosci. 2018 Oct 17;12:365. doi: 10.3389/fncel.2018.00365 (PMC6199958; doi:10.3389/fncel.2018.00365)

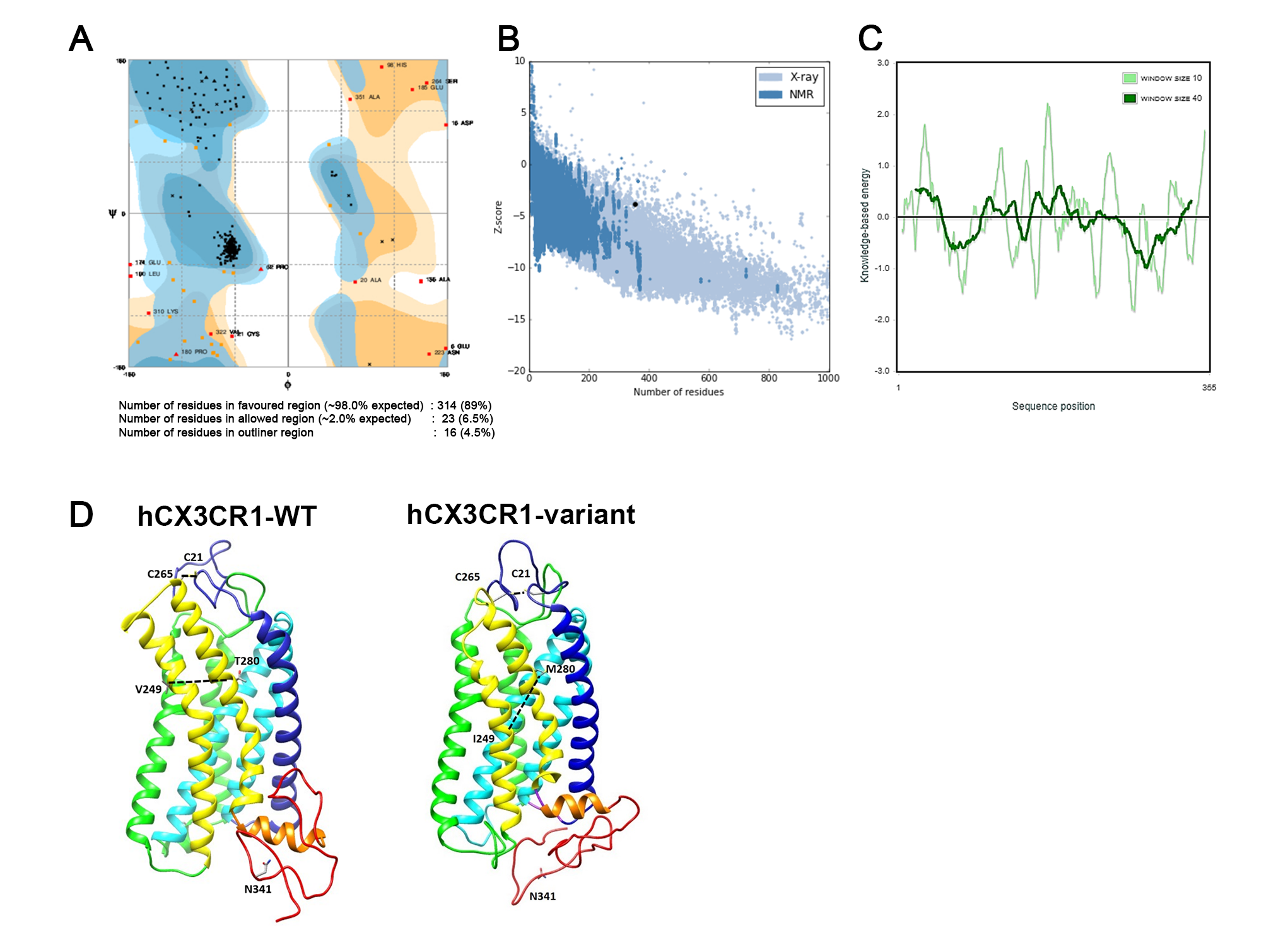

Supplement: FIGURE S1 — Quantitative validation of energetics and stereochemistry. (A) The proposed structural models were built using the prediction software Phyre2, Swiss-assessment structure suite that provides energetic values and the RAMPAGE software that characterizes the stereochemistry of the proposed model from measurement of dihedral angles with Ramachandran graphics, showing that 89% of the residues fall within the favored region, 6.5% in the allowed region and 4.5% in the outliner region, therefore 95.5% of the residues are within the degree of acceptance for the QMEAN6/Z score and Ramachandran plot. (B) Comparison of protein composition based on size given by Z-score values. The feasibility of the hypothetical model yields a value of -3.81, positioning the model within the X-ray and NMR area of the graph based on experimental data. (C) Energetic characterization based on fragments of amino acids along the primary sequence, first using a central amino acid and obtaining an energetic average in fragments of 10 and 40 amino acids (window size). Values above the ‘zero’ threshold are considered problematic and coincide within extracellular and intracellular loops that reflect flexible and disorganized areas that may function as the recognition site of the ligand fractalkine. Similarly, the QMEAN6 value obtained is derived from a combination of linear values including the energy of torsion angles, coupling energy of atom pairs, energy of solvation, and energy of the beta carbon, values of secondary structure and values of accessibility of solvent. The model is assigned a normalized value that estimates the reliability of the model with a range between 0–1. For the selected model the QMEAN6 value of 0.546 as a global parameter that is accepted when values are above 0.5. (D) The most reliable amino acid fragments coincide with the regions that are not mobile such as α-helices within the membrane (Figure 2). Loops are predicted with a disorganized orientation but with an extracellular [file Image_1.tif]

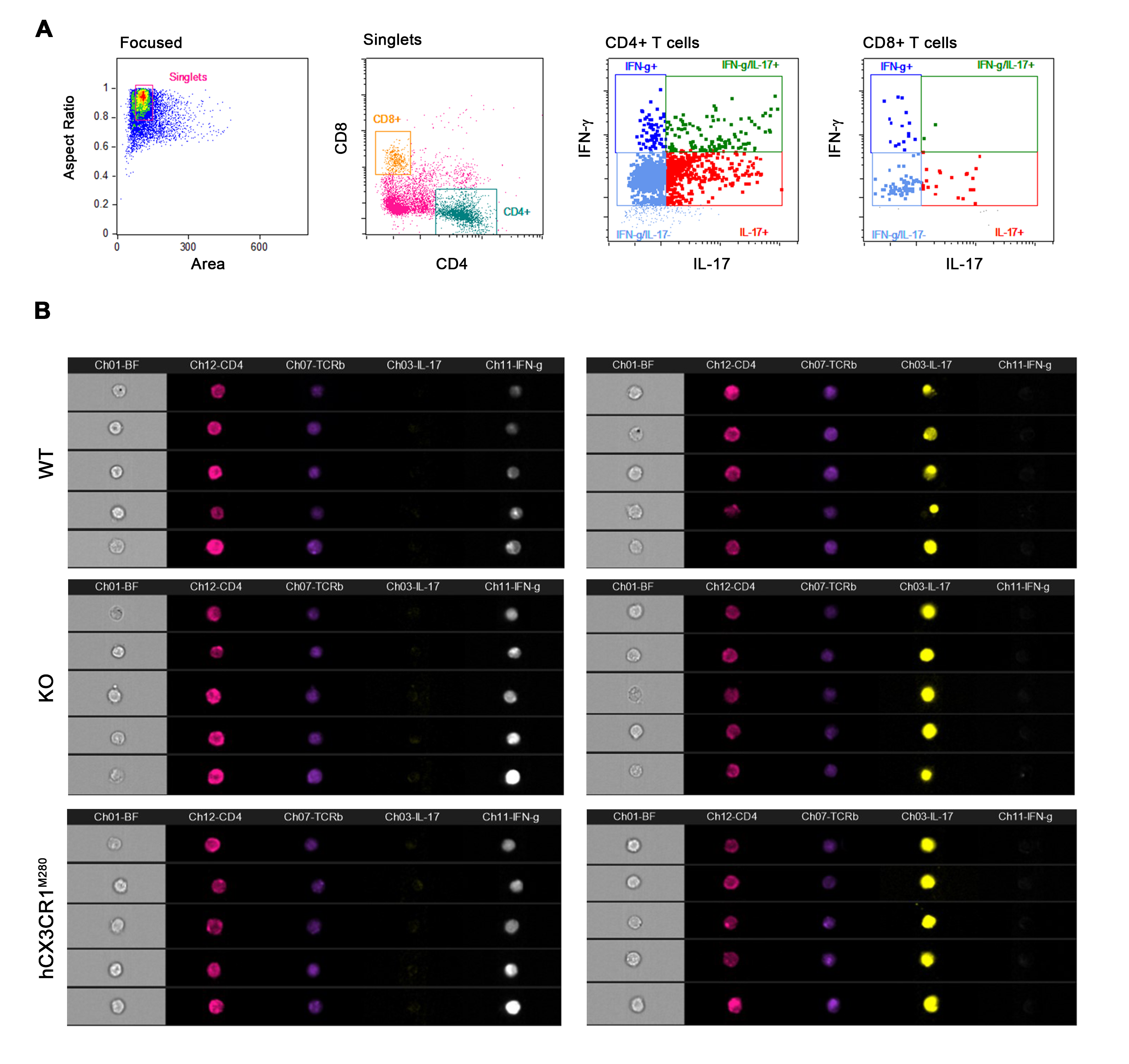

Supplement: FIGURE S2 — IL-17 and IFN-γ production by CD4+ and CD8+ T cells in EAE affected mice. (A) Brain and spinal cord mononuclear cells isolated over percoll gradients were subjected to imaging flow cytometry with gating strategy based on singlet population, followed by CD4 and CD8 T cell separation and IFN-γ and IL-17 discrimination. (B) Images show that double positive cells for IFN-γ/IL-17 were less abundant that the single positive counterparts and detected in similar proportions among the groups. [file Image_2.tif]

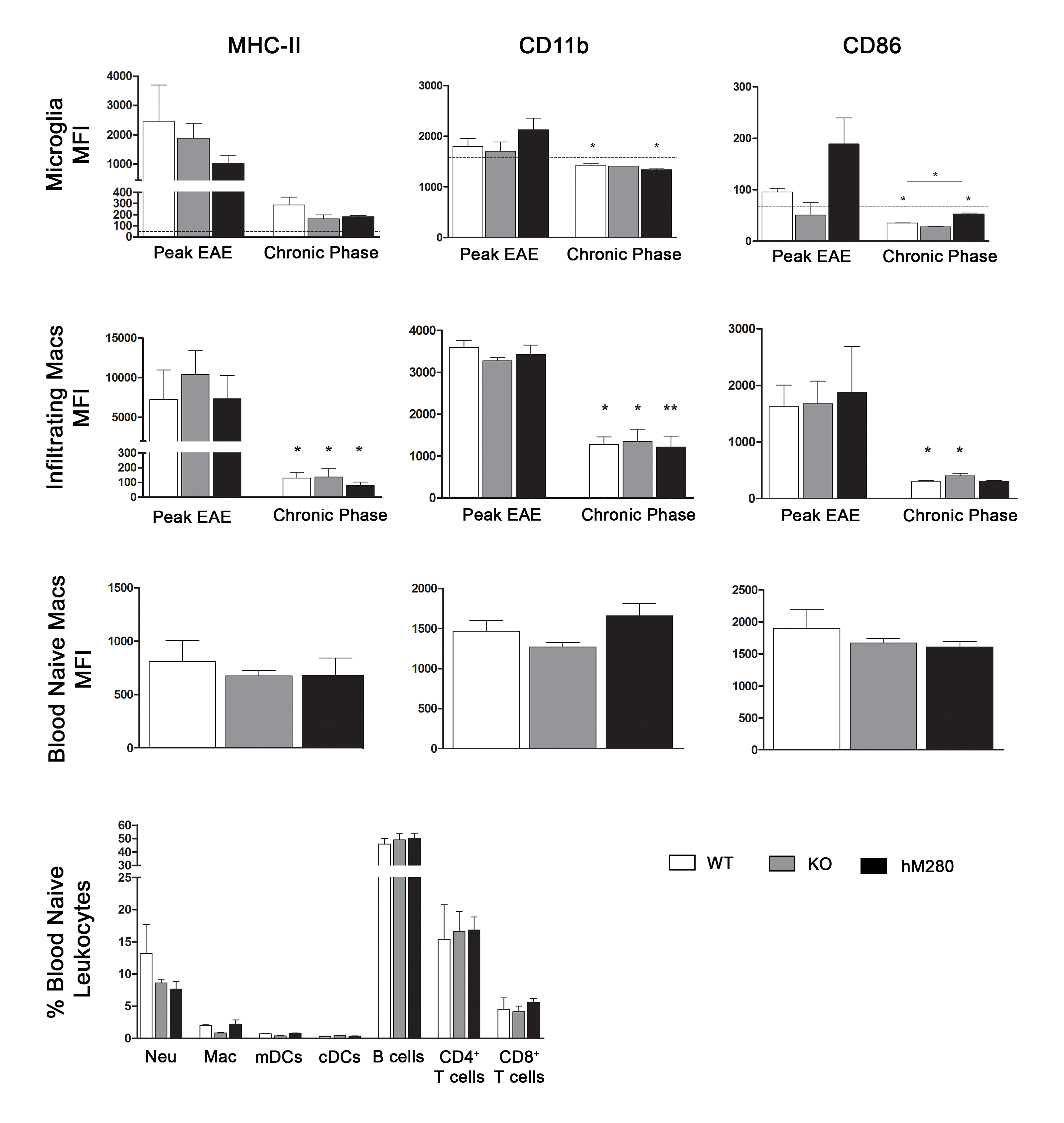

Supplement: FIGURE S3 — Signature profile of brain and blood leukocytes. Activation of brain and spinal cord microglia (CD45LoCD11b+) and infiltrating macrophages (CD45HiCD11b+Ly6C+) was assessed by flow cytometric assessment of median fluorescence intensity as a representation of abundancy of MHC-II (left panels), CD11b (middle panels) and CD86 (right panels) molecules. These activation markers were also evaluated in blood monocytes from naive mice. Microglia activated phenotype is characterized by increased MHC-II MFI at peak disease, whereas infiltrating monocytes also exhibit higher MFI for CD11b and co-stimulatory molecule CD86. Insertion of hCX3CR1I249/M280 does not appear to affect the peripheral immune populations (bottom panel). Presented p-values correspond to significant differences between mice of the same phenotype at peak vs. chronic disease, unless otherwise stated. [file Image_3.tif]
