# Supplementary material for: Role of the Fractalkine Receptor in CNS Autoimmune Inflammation: New Approach Utilizing a Mouse Model Expressing the Human CX3CR1I249/M280 Variant
Source: Front Cell Neurosci. 2018 Oct 17;12:365. doi: 10.3389/fncel.2018.00365 (PMC6199958; doi:10.3389/fncel.2018.00365)
Supplement: TABLE S1 — Genes significantly regulated in CX3CR1-WT microglia in comparison to naïve microglia. [file Table_1.pdf]

Supplementary table 1. Genes significantly regulated in CX3CR1-WT microglia in comparison to naïve microglia

Feature ID	EDGE test: Naive WT vs EAE WT, tagwise dispersions - Fold change	EDGE test: Naive WT vs EAE WT, tagwise dispersions - FDR p-value correction
Acod1	46.42833565	0.003264073
Adgrg5	41.20291804	0.021999806
Aldh1a1	-727.8535685	0.021827818
Apoc1	247.4224347	4.96909E-05
Apoe	14.14934089	1.50219E-07
Asic2	-421.5665306	0.049423977
Axl	12.14166721	1.35586E-05
B2m	3.836422997	0.005409951
Bst1	78.25577175	0.018153271
Bst2	4.9850156	0.009531337
C2	737.5165994	0.002601047
C3	29.63419339	0.000151029
C4b	124.8382866	3.25715E-18
Ccl12	6.697782541	0.001560794
Ccl22	124.0845603	0.035393055
Ccl5	157.9401629	1.04098E-06
Cd274	11.38619134	0.010579133
Cd300lf	83.80922329	0.045908832
Cd36	66.11795677	1.07188E-05
Cd40	77.02867197	0.001075592
Cd44	38.85127697	0.003100444
Cd52	7.672689551	4.61452E-05
Cd69	56.96913271	0.015521314
Cd72	44.28360937	0.000180499
Cd74	47.44411873	3.2984E-13
Cfb	179.178194	0.01448482
Ch25h	98.10668887	0.000376476
Clec7a	8.651251216	8.85024E-05
Cox6a2	466.892655	0.022868871
Cst7	25.51733679	2.93359E-06
Ctse	166.4879662	0.007775406
Cxcl10	23.55513507	0.049423977
Cxcl13	409.2953056	6.38742E-07
Cxcl9	65.63866343	2.5619E-08
Cyp4f18	65.25154719	0.001071233
Dao	-849.852604	0.038418429

Fabp5	22.29309831	0.044337431
Fcgr4	15.51124281	1.12707E-07
Fgf12	-778.6637843	0.01537098
Fn1	83.42280638	1.96407E-07
Gbp2	16.52397747	2.0344E-05
Gpnmb	39.59368334	0.002884519
Gpr132	273.9205664	0.00301626
H2-Aa	21.09461564	0.000293807
H2-Ab1	25.20991858	1.90945E-06
H2-D1	9.407315793	2.83364E-09
H2-Eb1	25.56614395	3.94878E-05
H2-K1	9.092625679	3.06064E-08
H2-Q4	11.19603825	1.35486E-06
H2-Q5	43.38733703	0.021827818
H2-Q6	35.41242522	5.46943E-11
H2-Q7	64.42173096	4.06471E-16
Hcar2	37.91678167	6.1017E-05
Helz2	10.31261839	0.015532083
I830127L07Rik	83.51084794	0.035393055
Ifi204	19.39986177	5.08597E-09
Ifi207	15.70865069	0.000818378
Ifi208	1222.836688	7.66898E-05
Ifi209	9.574852018	0.015521314
Ifi211	120.7192526	0.00225583
Ifi213	34.31328202	0.000818378
Ifi27l2a	42.84267596	0.000229644
Ifi30	5.310512972	0.003296385
Ifit2	23.05592545	0.002589007
Ifit3b	28.51282093	0.030752321
Ifitm1	123.3974362	0.040649404
Ifitm3	19.8409343	1.13549E-05
Iigp1	38.47428252	0.00052486
Ikbke	14.28026123	0.047942985
Il1b	16.71361402	0.027321294
Il1rn	18.52321611	0.046121152
Irf1	3.741503704	0.027540253
Irf7	21.83875089	4.76272E-07
Irgm1	4.933205167	0.006182711
Itgal	100.8064572	6.87682E-06
Itgax	16.55500799	2.0344E-05
Itgb7	31.28782006	0.012460397

Lcn2	54.03678828	0.000427696
Lgals3	15.01397355	0.016880783
Lgals3bp	7.593513	9.80342E-06
Lilrb4a_1	69.3718431	0.004835635
Lrg1	121.4640348	0.040649404
Ltf	21.03794115	0.030360975
Ly6a	21.91990691	0.001071233
Ly6c2	66.12001243	0.016610836
Ly6i	298.2465026	1.23884E-05
Lyz2	4.909142698	0.013987904
Met	196.3653003	0.007217368
Mlc1	-262.0348492	0.041726437
Mx1	10.96126654	0.002589007
Nlrc5	17.16896712	1.31546E-06
Oas1g	157.2373347	0.001845254
Oas2	79.36867491	4.21189E-08
Oas3	45.20675532	0.000135117
Oasl1	38.251093	0.001632048
Oasl2	34.62532183	3.06064E-08
Pak3	-162.7888796	0.046121152
Plac8	32.65830829	0.027321294
Plbd1	90.40245961	0.002947696
Prss12	-374.9697776	0.039656104
Pstpip1	22.29785353	0.009531337
Saa3	276.6951532	0.005049716
Sdc3	5.213358101	0.042528463
Sell	62.64677722	0.030752321
Serpina3f	62.0496299	0.015521314
Serpina3g	177.0435691	0.000135117
Slamf7	40.22238422	0.001845254
Slfn5	17.1728084	0.010688711
Sp100	10.01142606	0.004093103
Spp1	36.30080033	1.72466E-08
Stat1	4.978961881	0.003296385
Stat2	4.807617931	0.023986288
Synpr	-1129.486992	0.000134554
Tap1	5.550354857	0.001632048
Tspo	9.222448992	0.009153947
Vwa5b2	-928.0136311	0.007775406
Wfdc17	31.07699752	0.021827818
Wfdc21	107.1957911	0.021999806

Zbp1	41.14443151	3.83704E-05
