# Supplementary material for: Role of the Fractalkine Receptor in CNS Autoimmune Inflammation: New Approach Utilizing a Mouse Model Expressing the Human CX3CR1I249/M280 Variant
Source: Front Cell Neurosci. 2018 Oct 17;12:365. doi: 10.3389/fncel.2018.00365 (PMC6199958; doi:10.3389/fncel.2018.00365)
Supplement: TABLE S2 — Genes significantly regulated in CX3CR1-KO microglia in comparison to naïve microglia. [file Table_2.pdf]

Supplementary table 2. Genes significantly regulated in CX3CR1-KO microglia in comparison to naïve microglia

Feature ID	EDGE test: Naive KO vs EAE KO, tagwise dispersions - Fold change	EDGE test: Naive KO vs EAE KO, tagwise dispersions - FDR p-value correction
AB124611	20.09630628	0.047245996
Acod1	260.7586223	0.000453465
Anpep	91.5315585	0.013311803
Apoc1	148.0411873	0.004437756
Apoe	15.44472893	5.09321E-10
Axl	14.12422276	3.74821E-09
B2m	4.165428954	0.005894705
Bst2	5.23506258	0.008587286
C3	96.94858922	4.30301E-12
C4b	221.4688739	1.38816E-37
Ccl12	7.112333302	0.012173473
Ccl5	80.10457822	4.59653E-06
Ccl8	2003.549388	0.019432002
Cd244	24.83046515	0.029479876
Cd274	10.07436771	0.000226175
Cd36	269.9808563	3.1489E-10
Cd52	7.766472074	9.66403E-05
Cd63	4.640620251	0.025592175
Cd69	454.5580307	0.00273643
Cd72	22.38286684	3.88955E-06
Cd74	61.72627129	2.03761E-17
Ch25h	52.87536445	0.029479876
Clec7a	5.955995301	0.001237197
Colec12	156.7656095	0.033879685
Cpd	4.563622499	0.01067625
Csf1	5.222986698	0.013786388
Cst7	61.8656254	4.30301E-12
Cxcl13	582.2233531	3.17823E-05
Cxcl16	4.329398095	0.033879685
Cxcl9	108.45107	8.59606E-11
Cybb	24.34574505	1.31696E-08
Ddx3y	-44.01741078	0.004803987
Eif2s3y	-101.5332101	1.9215E-05
Fcgr4	22.79272391	2.67859E-08
Fn1	253.7248551	6.51701E-29
Fscn1	-4.615052432	0.002630799

Fxyd5	12.25353153	0.013786388
Gbp2	62.55668789	1.00887E-15
Gbp3	7.798811405	0.001111857
Gbp4	389.4126068	0.001667743
Gbp5	10.45360107	2.15967E-06
Gbp7	4.613146429	0.011777146
Gbp8	116.5825871	8.81247E-08
Gm13889	-267.4853	0.033879685
Gpnmb	36.85054859	0.016179639
Gpx3	127.47965	0.006674395
H2-Aa	56.60502933	4.50628E-11
H2-Ab1	39.66175788	1.09265E-12
H2-D1	10.82485519	6.89119E-09
H2-Eb1	41.52182103	7.38286E-09
H2-K1	10.42906451	8.81247E-08
H2-Q4	14.83932141	2.17635E-09
H2-Q5	30.93457222	0.004437756
H2-Q6	79.18586253	2.74346E-21
H2-Q7	165.7486137	6.51701E-29
Hcar2	17.26965625	1.50785E-06
Helz2	15.75281589	5.54949E-05
Hpca	-1630.102991	0.007489575
Hpse	118.4152655	0.020885269
Ifi204	29.15693611	7.16913E-07
Ifi206	83.46415473	0.049659848
Ifi207	74.16787603	0.001443035
Ifi211	1170.630612	0.027359084
Ifi213	68.71524798	0.033879685
Ifi27l2a	22.16433866	6.62048E-06
Ifi30	4.785598385	0.008587286
Ifit2	11.82835226	0.000144565
Ifit3	10.08362249	0.048791512
Ifitm3	21.49223913	1.08081E-05
Iigp1	528.4575803	7.19072E-14
Ikbke	38.66923643	0.002396087
Il12rb1	904.1486785	0.000110934
Il18bp	22.96579593	0.003365097
Irf1	4.727256723	0.003378048
Irf7	12.51692318	3.09476E-07
Irgm1	6.521215634	7.19634E-05
Itgal	298.6301064	3.14009E-11

Itgax	8.825649271	4.24816E-05
Kdm5d	-808.5948652	2.82096E-05
Klhdc8b	-6.52907441	0.033864307
Lgals3bp	9.746958912	1.88558E-08
Lilrb4a_1	102.3076663	0.040653499
Ly6a	24.25789378	1.9215E-05
Ly6i	192.9345552	0.020885269
Lyz2	4.928532202	0.001941294
mt-Co2	237.1567972	0.021791824
Mx1	11.13227381	0.000705084
Nampt	5.637520356	0.005913357
Nlrc5	35.63613803	7.20104E-17
Oas2	36.09522957	3.58902E-11
Oas3	428.4968712	7.74005E-06
Oasl2	14.31314189	2.53684E-06
Parp14	3.744398029	0.047245996
Pdyn	99.99078267	0.040653499
Rnf213	5.503395543	0.005637812
Saa3	276.0516938	0.000337351
Sdc3	4.950237201	0.003306787
Serpina3f	882.9603379	0.001177149
Serpina3g	2467.963182	0.044159766
Slamf8	6.67815243	0.028629926
Slc2a6	12.68230015	0.000458052
Slfn5	19.89593565	0.009494618
Spp1	25.8686246	1.76255E-07
Stat1	7.597142619	9.37629E-06
Stat2	7.039883704	1.32967E-05
Tap1	7.561940218	3.05045E-06
Treml2	7.300362889	0.030809105
Tspo	9.610633993	0.011015747
Uty	-226.3226412	0.019432002
Xdh	53.58716972	0.000196412
Zbp1	94.12838832	5.189E-15
